# Pulmonary Artery Intimal Sarcoma Diagnosed Preoperatively by Endovascular Biopsy and Treated via Right Pneumonectomy and Pulmonary Arterioplasty

**DOI:** 10.1155/2021/5573869

**Published:** 2021-06-27

**Authors:** Daiki Kato, Shohei Mori, Eriko Harada, Rintaro Shigemori, Takamasa Shibazaki, Hideki Matsudaira, Jun Hirano, Hirokazu Ashida, Hiroko Kimura, Michio Yoshitake, Takashi Kunihara, Takashi Ohtsuka

**Affiliations:** ^1^Department of Surgery, Division of Thoracic Surgery, The Jikei University, School of Medicine, 3-25-8 Nishishinbashi, Minatoku, Tokyo 105-0003, Japan; ^2^Department of Radiology, The Jikei University, School of Medicine, Japan; ^3^Department of Pathology, The Jikei University, School of Medicine, Japan; ^4^Department of Cardiac Surgery, The Jikei University, School of Medicine, Japan

## Abstract

*Introduction*. Intimal sarcoma is a very rare tumor arising within the intima of the pulmonary artery. Preoperative diagnosis of pulmonary artery sarcoma is difficult, and the tumor is sometimes misdiagnosed as pulmonary thromboembolism. We report a case of pulmonary artery intimal sarcoma successfully diagnosed by preoperative endovascular biopsy and treated via right pneumonectomy and pulmonary arterioplasty. *Presentation of a Case*. A 72-year-old woman was referred to our hospital with a low-attenuation defect in the lumen of the right main pulmonary artery by computed tomography. Pulmonary artery thromboembolism was suspected, and anticoagulation therapy was administered. However, the defect in the pulmonary artery did not improve. Endovascular catheter aspiration biopsy was performed. Histological examination revealed pulmonary artery sarcoma. The patient was treated with right pneumonectomy and arterioplasty with the use of cardiopulmonary bypass. *Discussion*. Preoperative biopsy by endovascular catheter is worth considering for a patient with a tumor in the pulmonary artery and can help in planning treatment strategies.

## 1. Introduction

Pulmonary artery sarcoma is a very rare tumor that is sometimes misdiagnosed as pulmonary thromboembolism. Only a few cases have been preoperatively diagnosed as pulmonary sarcoma. In this report, we present a case in which pulmonary artery sarcoma was diagnosed by transvenous endovascular catheter biopsy preoperatively, and right pneumonectomy with arterioplasty was successfully performed.

## 2. Case Presentation

**Figure 1:**
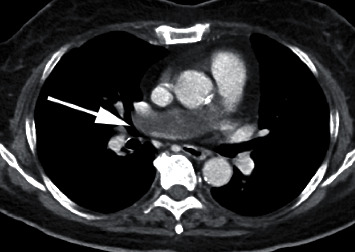
A contrast-enhanced computed tomography scan showing a tumor (arrow) in the right main pulmonary artery.

**Figure 2:**
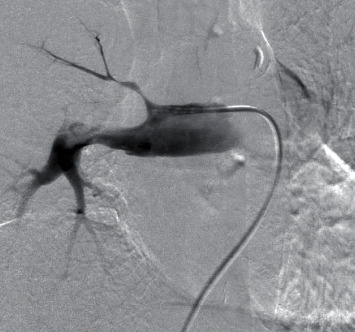
A pulmonary angiogram revealing tumor of the right main pulmonary artery. The catheter is attached to the filling defect, and vacuum suction is performed.

A 72-year-old woman presented with a 2-month history of mild dyspnea on exertion. A computed tomography (CT) scan showed a low-attenuation defect in the lumen of the right main pulmonary artery (Figure 1). Pulmonary artery thromboembolism was suspected. Anticoagulation therapy with apixaban was administered; however, the defect in the pulmonary artery did not improve after 4 weeks of anticoagulation treatment. A positron emission tomography (PET) scan revealed the uptake of fluorine-18 fluorodeoxyglucose (FDG) in the intraluminal defect with a maximum standardized uptake value (SUVmax) of 4.35, suggesting a malignant tumor. To confirm the diagnosis and selection of a treatment, we performed endovascular catheter aspiration biopsy of intravascular mass using a 6 Fr Envoy catheter (Codman, Raynham, Massachusetts, USA). Vacuum suction of the mass was performed several times by careful manipulation (Figure 2). Histological examination revealed pulmonary artery sarcoma. No distant metastasis was identified. A right pneumonectomy with arterioplasty of the pulmonary arterial bifurcation with the use of cardiopulmonary bypass was planned.

**Figure 3:**
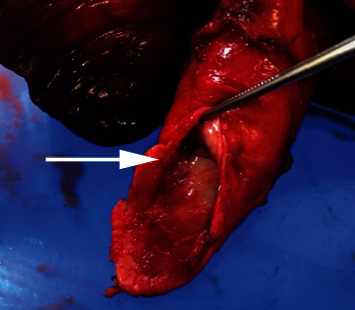
An occlusive tumor in the main pulmonary artery (arrow) of resected specimen.

**Figure 4:**
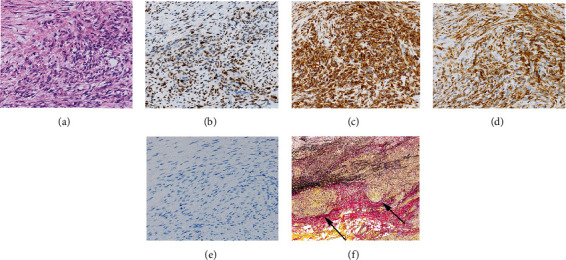
Histopathology of intimal sarcoma (×200). (a) Hematoxylin and eosin stain showing atypical spindle-shaped tumor cells. (b) Immunohistochemical staining showing positive staining for murine double minute type 2 homologue. (c) Immunohistochemical staining showing positive staining for vimentin. (d) Immunohistochemical staining showing positive staining for alpha smooth muscle actin. (e) Immunohistochemical staining showing negative staining for desmin. (f) Elastica-van-Gieson staining showed tumor cells invading outside pulmonary artery. Arrows indicate that the wall of pulmonary artery was destroyed by tumor invasion.

Through median sternotomy, the surgery was performed. The tumor completely filled the right main pulmonary artery. No invasion to extravascular tissues was identified. Cardiopulmonary bypass was established with standard techniques using cannulas placed in the ascending aorta and the right atrial appendage. The main pulmonary artery was incised. The solid tumor filled the right main pulmonary artery and extended to the main pulmonary artery trunk. The right pulmonary artery and a part of the main pulmonary artery from the supravalvular to the left main pulmonary artery were resected. Macroscopically, the surgical margin was negative. The main pulmonary artery trunk and left pulmonary artery were reconstructed with bovine pericardium. The patient was successfully weaned from the cardiopulmonary bypass, and a right pneumonectomy was performed. The operation time was 429 min, and the cardiopulmonary bypass time was 73 min. The blood loss was 920 mL. The gross specimen showed that yellowish solid mass, 9 × 5 × 3 cm in size, was occluding the right main pulmonary artery (Figure 3). Histological examination of the tumor revealed intimal sarcoma. Immunohistochemical staining was positive for vimentin, murine double minute type 2 homologue, and alpha smooth muscle actin (Figure 4), negative for desmin, factor VIII, CD34, and CD31. Tumor cells were invading outside pulmonary artery. A few tumor cells were found at the resected margin of the left distal pulmonary artery. The patient was discharged 28 days postoperatively without any complications. The patient received radiotherapy postoperatively. A series of subsequent chest CT scans did not show signs of recurrence of the tumor 6 months postoperatively.

## 3. Discussion

Pulmonary artery intimal sarcoma occurs in the proximal elastic arteries from the level of the pulmonary valve to the lobar branches. In most cases, intimal pulmonary sarcoma is diagnosed as an advanced disease spreading to the adjacent lung and heart; this tumor is sometimes found with distant metastasis to the lung, brain, and adrenal glands [1]. Preoperative diagnosis of pulmonary artery intimal sarcoma is challenging, and many cases are misdiagnosed as pulmonary emboli or chronic thromboembolic pulmonary hypertension [2, 3]. Contrast-enhanced CT scan often shows a heterogeneous low-density filling defect of the pulmonary artery. FDG-PET can be used to differentiate thrombi from pulmonary artery intimal sarcoma. Ito et al. reported that intimal sarcoma of the pulmonary artery had a higher SUVmax than the thrombus (7.63 and 2.31, respectively) [4]. There are some reports of preoperative biopsy, including CT-guided transthoracic biopsy and transbronchial biopsy [1]; however, increased bleeding has been reported. Although reports on endovascular biopsy are few, endovascular biopsy seems to be a safe and promising approach for intrapulmonary artery tumors and should be considered before surgical intervention. Preoperative histological diagnosis can help select treatment options. In case of surgery, it can avoid an intraoperative frozen section that would prolong the cardiopulmonary bypass time. Endovascular catheter biopsy is a direct approach to the tumor and can minimize the risk of bleeding, pneumothorax, or implantation related to transthoracic CT-guided biopsy.

Intimal sarcoma commonly arises from intimal layer of large blood vessels, such as heart, pulmonary artery, aorta, and superior vena cava, whereas peripheral arterial sarcomas are exceptionally rare, with only a few reported cases of intimal sarcoma. One report is the intimal sarcoma in common femoral artery, and the other is in a superficial femoral artery [5, 6]. Those sarcomas are characterized by an endoluminal growth that progresses to vessel obstruction or embolic seeding of distal sites.

The treatment options for intimal pulmonary artery sarcoma include surgery, chemotherapy, and radiation. Multimodal treatment with chemotherapy and radiotherapy might result in a favorable outcome [7]. However, a chemotherapy regimen for intimal sarcoma has not been defined because of the rarity of the disease. Complete resection is the only factor for prolonged survival [7]. Although no macroscopic evidence of tumor was found at the resection margin in the present case, a few tumor cells were found at the resected margin of the left distal pulmonary artery. This patient underwent radiotherapy after the surgery at resection margins, although postoperative radiation was not established.

## 4. Conclusions

In conclusion, when a tumor in the pulmonary artery remains unchanged despite anticoagulation treatment, percutaneous transvenous biopsy of the tumor should be performed. Early diagnosis of pulmonary artery sarcoma can lead to surgical resection, which can contribute to long-term survival.

## Abbreviations

CT: Computed tomography.
